# A mixed methods evaluation of the breastfeeding memory aide CHINS

**DOI:** 10.1111/mcn.13704

**Published:** 2024-07-19

**Authors:** Lynette Shotton, Tracy Collins, Reinie Cordier, Fadzai Chikwava, Mary Steen

**Affiliations:** ^1^ Department of Social Work, Education and Community Well‐being Northumbria University Newcastle Upon Tyne UK; ^2^ Curtin School of Population Health Curtin University Bentley Western Australia Australia

**Keywords:** breastfeeding, breastfeeding tool, CHINS, mixed methods, normalisation process theory, positioning

## Abstract

Breastfeeding rates remain persistently low in the United Kingdom (UK) despite wide‐scale rollout of UNICEF Baby Friendly Initiative training and accreditation. More must be done to ensure breastfeeding practitioners can provide effective support. The memory aide CHINS (Close, Head free, In‐line, Nose to Nipple and Sustainable) could help practitioners remember, recall, and apply breastfeeding theory in practice and this paper presents a UK evaluation of its impact. A concurrent, convergent mixed methods approach was adopted using Normalisation Process Theory (NPT) as an overarching framework. An online survey targeted breastfeeding practitioners and academics from the UK (*n* = 115). A sub‐set (*n* = 16) of respondents took part in qualitative focus groups. Survey data was subjected to descriptive and inferential statistical analysis, and the focus group data was analysed, using NPT. CHINS is widely used in breastfeeding education and practice largely because of its simplicity and ease of integration in everyday practice, as well as its sustained inclusion in UNICEF Baby Friendly Initiative training. CHINS has introduced a standardised approach to the principles of positioning for effective breastfeeding. Doing so has helped address inconsistencies and poor practice in this area, and CHINS plays a role in assisting practitioners in building confidence in their breastfeeding practice. More needs to be done to ensure the breastfeeding workforce develop and maintain the requisite skills to promote and support breastfeeding, including the role of memory aides such as CHINS in achieving this.

## Key messages

- Breastfeeding is a key public health priority.
- Rates of breastfeeding vary across the United Kingdom.
- The breastfeeding workforce needs adequate training.
- The memory aide CHINS is widely used, but this is the first formal evaluation of its value in promoting principles of positioning for effective breastfeeding.

## Graphical abstract

This paper presents findings of a mixed methods evaluation of the mnemonic Close, Head free, In‐line, Nose to nipple, Sustainable (CHINS).

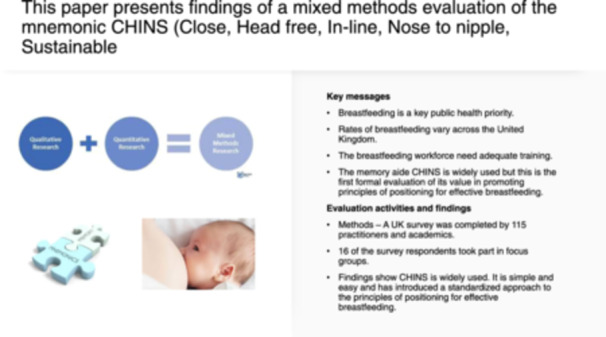

## 1 INTRODUCTION

Breastfeeding is a key public health priority that reduces the risk of gastrointestinal, respiratory and ear infections and hospital admissions, protects against obesity, and positively impacts intelligence (World Health Organization, 2023b). For mothers, breastfeeding protects against breast and ovarian cancer and type 2 diabetes (Royal College of Paediatrics and Child Health, 2022). Alongside these health benefits, breastfeeding is considered the most environmentally friendly and sustainable form of infant feeding (Smith et al., 2022). UNICEF estimates that even moderate increases in breastfeeding rates would save up to £50 million each year in NHS expenditure (UNICEF, 2023).

Over the last decade, the prevalence of exclusive breastfeeding during the first months of life has increased by 10% to 48% globally (World Health Organization, 2023a). However, the United Kingdom (UK) continues to have some of the lowest breastfeeding rates in the world. While the proportion of new mothers initiating breastfeeding has increased from 66% in 2005/6 to 74% in 2010/11, remaining stable until 2016/17, this has dropped slightly to 72%, and rates of babies who are partially or fully breastfed dropped significantly at 6–8 weeks with 2021/22 data showing rates of 49%. Only 34% of babies receive some breastmilk at 6 months, compared with 49% in the United States and 71% in Norway (Royal College of Paediatrics and Child Health, 2022).

Understanding the reasons behind low breastfeeding rates is complex, but there is increasing recognition of the widening socioeconomic inequalities associated with infant feeding (Baker et al., 2023). Indeed, a recent UK study suggests that the neighbourhood directly influences initiation. In areas of socioeconomic deprivation, mothers are much less likely to initiate and continue to breastfeed, and this relates to social norms, peers, and social support, as well as the availability of and access to health services (Peregrino et al., 2018). The lack of information and effective practical support are cited as barriers (Royal College of Paediatrics and Child Health, 2022), and it is increasingly recognised that widespread and consistent support from key public health services in local communities is needed (UNICEF, 2023). As such, healthcare professionals providing support and care for mothers and infants need the knowledge and skills to promote, protect and support breastfeeding to enable women to commence and continue breastfeeding for as long as they want to (Royal College of Nursing, 2019a).

While there has been a wide‐scale roll‐out of UNICEF Baby Friendly training and accreditation across the United Kingdom since 1984 (UNICEF, 2023), there is evidence to suggest that the uptake of training and accreditation of maternity and community services such as midwifery and health visiting is variable, leading to differing levels of knowledge and skill (Local Maternity System Public Health Prevention (2019). Indeed, Yang et al. (2018) suggest that healthcare professionals and students do not always feel adequately trained to support breastfeeding. While UNICEF Baby Friendly Training is considered one of the best approaches to evidence‐based training (NHS, 2019), it is increasingly clear that other approaches are needed, and a key recommendation in the Lancet Series on breastfeeding calls for a marked expansion in health professional training on breastfeeding and young child nutrition (Baker et al., 2023). Memory aides are widely used in healthcare practice and can help practitioners to remember key theories and recall them, as well as provide structure and guidance for practice (Maheshwari & Kaur, 2019). In view of this, the memory aide for principles of positioning for effective breastfeeding Close, Head free, In‐line, Nose to Nipple, Sustainable (CHINS) could play an important role in strengthening existing work, offering an easy‐to‐explain and share, simple but effective tool to help practitioners understand, remember, recall and apply breastfeeding theory in their routine practice.

### 1.1 Background

The memory aide CHINS is a first‐letter mnemonic developed in 2010 by Harland (2011) to help practitioners remember, retain and recall evidence‐based theory about the principles of positioning babies for effective breastfeeding in a simple and structured manner. Each of the first letters relates to a word or phrase outlining a principle for effective positioning. When delivered as part of breastfeeding education and training, the mnemonic should be accompanied by an explanation and rationale for each principle so that practitioners know how to support positioning for effective breastfeeding and why. Table 1 provides an overview of CHINS.

**Table 1:** An overview of CHINS.

**C** lose: babies need to be close to their mothers so they can scoop enough breast into their mouths. Ensure both the mother's and baby's clothing and hands are not in the way.
**H** ead free: when attaching to the breast, babies tilt their heads back. This allows the chin to lead as it comes to the breast. Even a finger on the back of the baby's head will restrict this important movement.
**I** n line: the baby's head and body should be in alignment so they do not have to twist their neck, which would make feeding and swallowing difficult.
**N** ose to nipple: with the mother's nipple resting below the baby's nose, they will begin to root. As the baby tilts its head back, the nipple will slip under its top lip upwards and backward to rest between the hard and soft palate. Nose to the nipple is the starting point for effective attachment.
**S** ustainable: mothers need to be comfortable and relaxed and in a position that suits them best.

CHINS has been used within UNICEF Baby Friendly Initiative training since 2010 and can be found in a range of National Health Service (NHS), Local Authority and Public Health literature across the United Kingdom and increasingly in publications internationally, but until now, it has not been subject to formal evaluation. Therefore, this study, funded by the Burdett Trust for Nursing, presents the findings of a UK mixed methods evaluation of the memory aide CHINS.

## 2 METHODS

### 2.1 Underpinning theory

#### 2.1.1 Normalisation process theory

Normalisation Process Theory (NPT) provides a theoretical framework to understand how interventions, such as CHINS, are implemented, embedded, and integrated into healthcare settings (May et al., 2015). While initially developed as an applied theoretical model, this framework sought to assist the understanding and evaluation of the factors that enable or restrict the incorporation of healthcare innovations into routine practice. NPT was further developed into the current NPT, incorporating constructs that seek to understand how stakeholders understand, make sense of, engage with, and appraise innovations (Huddlestone et al., 2020). In this study, an adapted version of NPT (as shown in Table 2) was used to guide survey and focus group questions and as a theoretical lens to analyse the data drawing on the four constructs of coherence, cognitive participation, collective action and reflexive monitoring.

**Table 2:** Adapted NPT constructs used to guide this mixed methods study.

**Coherence**: Describe the factors that drive or inhibit the routine implementation of CHINS.	Does the purpose of CHINS make sense to clinicians and strategic leaders? Is it clear how to use CHINS? Has it brought about change/improvements to practice?
**Cognitive participation** Describe how CHINS is promoted and sustained in organisational settings.	How do practitioners drive forward CHINS? Why do practitioners drive forward CHINS?
**Collective action** Describe the individual and organisational implementation and promotion of CHINS.	Do practitioners have the right skills to implement CHINS? Do local/national policies, procedures and ways of working support the implementation of CHINS?
**Reflexive monitoring** Describe how CHINS is appraised and modified by staff in organisational settings.	Can practitioners judge the impact and effectiveness of CHINS? Can practitioners adapt/modify CHINS based on evaluation and experience?

### 2.2 Primary research question

#### 2.2.1 How is CHINS used and appraised by breastfeeding practitioners and academics in the United Kingdom?

2.2.2

**Aim and objectives**

*Aim*: to conduct a mixed methods evaluation of the memory aide CHINS among breastfeeding practitioners and academics in the United Kingdom.

*Objectives*:

1. A UK‐wide survey was conducted to understand why and how breastfeeding practitioners and academics use CHINS and how this is influenced by role, experience and level of training.
2. To explore participants' views and experience of using CHINS in depth through a series of focus groups with a subset of survey respondents.
3. To combine the findings to make judgements regarding the value of CHINS in breastfeeding education and practice.

### 2.3 A mixed methods approach

Consistent with a pragmatic paradigm (Kaushik & Walsh, 2019), a mixed methods approach was adopted to strengthen this first evaluation of the memory aide CHINS (Creamer, 2017). Here, the aim was not only to provide a comprehensive evaluation of CHINS through a UK‐wide survey but also to obtain detailed insight via focus group interviews (Doyle et al., 2016). In this study, the survey was released first, but as soon as responses were obtained, the process of identifying focus group participants began. This is broadly aligned to a concurrent design, where survey and focus group data were collected in the same time period and, importantly, independently of each other, where the findings of the survey did not influence the focus group data collection activities as would occur in a sequential approach (Creswell & Clark, 2017; Teddlie, 2009). Both methods aimed to answer the primary research question using an overarching deductive framework based on the four key concepts of the Normalisation Process Theory (Guest & Flemming, 2015).

**Figure 1:**
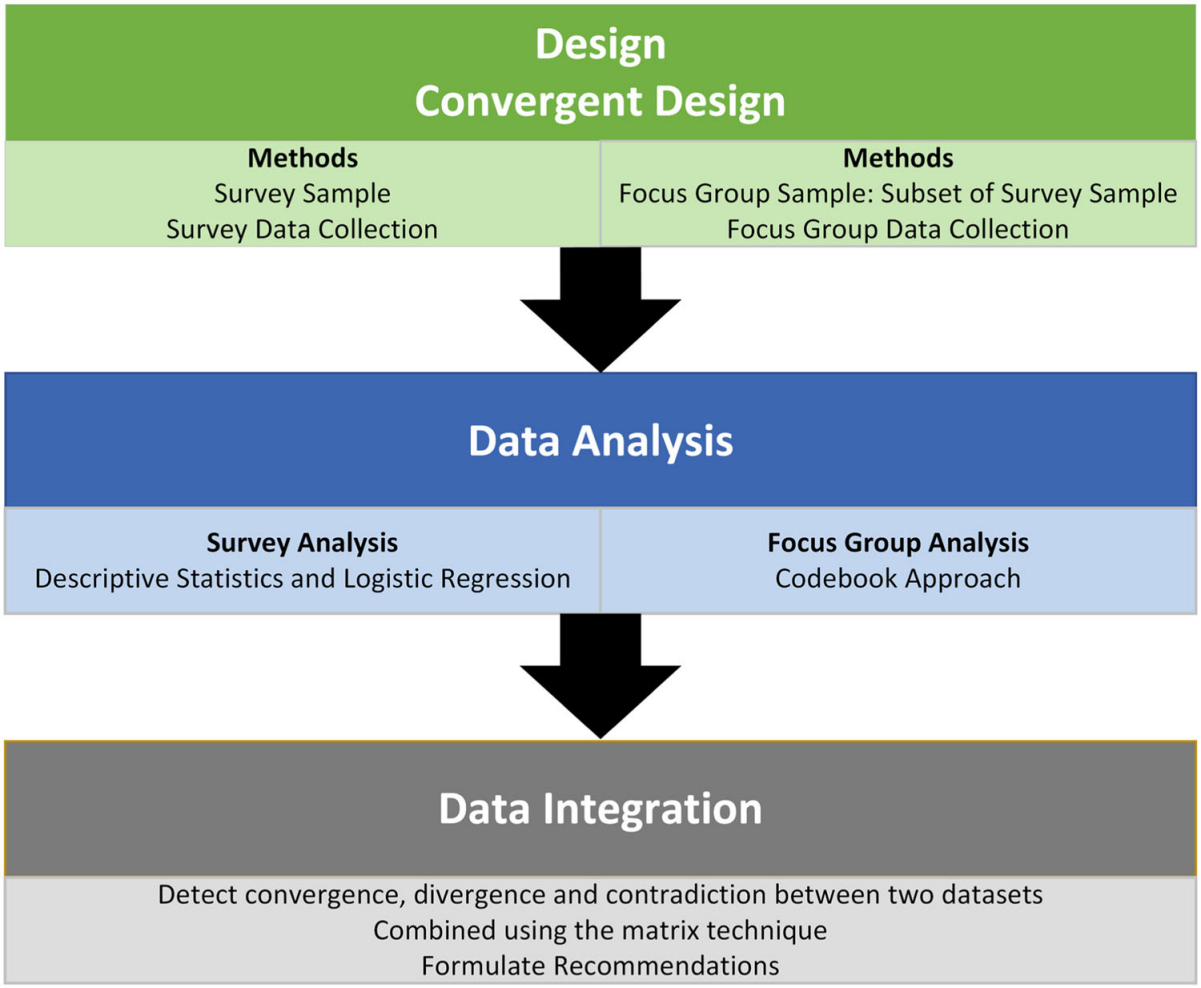
Levels of integration in this mixed methods evaluation.

To visualise the research activities and evaluation of this mixed methods study, see Figure 1.

### 2.4 Ethical considerations

Ethical approval was obtained (study ID 40808) before recruitment. The online survey required participants to read information about the study and provide consent before proceeding to the survey questions. Responses were anonymised, and participation was voluntary. Participation in the focus groups was also voluntary; informed consent was obtained and audio‐recorded at the beginning of each focus group. Interview transcripts were anonymised and stored on a password‐protected, secure university server. Audio recordings were destroyed following transcription.

### 2.5 Online survey

An online survey (see Supporting Information S1: File 1) was developed drawing on NPT (May et al., 2015) to collect socio‐demographic and opinion‐based responses to understand the extent to which CHINS had been integrated into practice and the reasons why. The first questions focus on capturing background information about the participants and their experience of breastfeeding training and practice. Question 28 specifically asked whether participants had heard of CHINS. If they had, they were directed to questions focused on NPT. They were directed to an overview of CHINS, and questions focused on its perceived value if they had not. The final set of questions was specific to NPT constructs and based on the NoMAD instrument, which offers a set of NPT questions that can be adapted to conduct quantitative research about implementation work (May et al., 2015; Rapley et al., 2018). The NOMAD tool was developed as a tool to measure the constructs of normalisation process theory, and its development included a range of quality appraisal processes and expert critique before being validated with 831 surveys of participants implementing six different implementation projects in a range of settings (May et al., 2015). The complete survey was piloted with three senior research assistants who have experience conducting research using NPT and mixed methods approaches in the field of health and social care but were not involved in the study. This ensured the questions were clear, the survey flowed well, and the survey could be conducted efficiently.

Recruitment to the survey was purposive, targeting healthcare professionals and university academics with a key role in promoting and supporting breastfeeding in the United Kingdom using a digital flier that was shared via UNICEF BFI networks, The National Infant Feeding Network, Higher Education Institutions (HEIs) that train midwives and health visitors, and social media to maximise response rate. The survey was completed by 115 respondents comprised of university academics, healthcare professionals and breastfeeding practitioners from across the United Kingdom.

Table 3 describes the sample. Almost all (99%) of the respondents were females and were of White background. The top three main professions were midwives (47%), university academics (21%), health visitors (12%), and UNICEF BFI educators (12%). The data reflects that some respondents had multiple professions. The main employer was NHS (67%), followed by university (25%), which was consistent with the type of profession mentioned. Most respondents had been employed for less than 10 years (68%), and a substantial number had been employed for longer than 10 years (42%). More than 80% had attained at least a degree‐level qualification.

**Table 3:** Characteristics of the survey population.

Characteristics	*N* (115)	%
Sex		
Female	114	99.1
Male	0	0.0
Not mentioned	1	0.9
Profession		
University academic	24	21.1
Midwife	54	47.4
Health visitor	14	12.3
UNICEF BFI Educator	14	12.3
Student	11	9.6
Family support worker	8	7.0
Neonatal nurse	4	3.5
Current Employer		
University	29	25.2
NHS	77	67.0
Local Authority	10	8.7
Student	5	4.4
Other	8	7.0
Duration of employment		
0–2 years	33	28.7
3–5 years	17	14.8
6–10 years	17	14.8
More than 10 years	48	41.7
Duration as a qualified clinician		
0–2 years	18	16.4
3–5 years	7	6.4
6–10 years	25	22.7
More than 10 years	60	54.6
Highest academic/professional qualification		
Diploma	14	12.2
Degree (BA/BSc)	67	58.3
Masters	23	20
Doctorate	5	4.4
Other	6	5.2
Ethnicity		
White	110	95.7
Other (Asian/African)	5	4.3

### 2.6 Focus group data collection

The focus group participants were a subset of the survey sample, identified by a question in the survey asking if they would be willing to participate. Thirty of the 115 survey respondents indicated they would be willing to participate; however, many cancelled, and despite being offered alternative dates, only 16 took part. Five focus groups were held in total, with between 2 and 6 participants. The participants were from a range of professional backgrounds: infant feeding leads (*n* = 4); midwife/midwifery lead (*n* = 2); specialist health visitor (*n* = 1); infant feeding co‐ordinator (*n* = 2); midwifery academic (*n* = 4); clinical manager 0‐19 service (*n* = 1); midwifery student (*n* = 1); Association of Breastfeeding Mothers Counsellor (*n* = 1). All were female and from a White British/Scottish background. The exact region where participants worked is not provided in this paper to protect anonymity, given the specialist nature of some of the roles. Still, there was representation from across England (*n* = 14) and Scotland (*n* = 2).

The lead author facilitated the focus groups via Microsoft Teams to accommodate attendance across the United Kingdom. Consistent with the convergent design described earlier, the focus groups aimed to capture more detailed insight into the participants' experiences of using CHINS, where Normalisation Process Theory informed questions and by adopting a more inductive approach (see Supporting information S1: File 2). The three research assistants who reviewed the survey reviewed the focus group questions for clarity.

### 2.7 Focus group data analysis

Focus groups were transcribed verbatim by a university‐approved independent transcriber. Consistent with the theoretical framework, NPT, used to guide this study, qualitative data analysis followed the codebook approach outlined by Crabtree and Miller (1992). This approach utilises a coding structure based on a priori interests, namely the four constructs of NPT that were applied to the data set. The coding was undertaken independently by two research team members (redacted for peer review) using a simple approach in Microsoft Word, whereby relevant text sections were highlighted and sorted to the a priori codes. Collectively, the researchers compared findings and agreed on the final content for each a priori code.

## 3 RESULTS

### 3.1 Survey results

The analysis of survey data comprised participants' characteristics (demographic, professional development and training; independent variables) and CHINS characteristics (dependent variables) as described below.

### 3.2 Participants' characteristics

Demographic characteristics included participants' gender, profession, current employer, length of employment, duration of working as a qualified clinician, highest academic qualification, and ethnicity. Professional development and training characteristics included training provider, duration since training was completed, frequency of engaging in professional development, frequency of education around breastfeeding, and key drivers in promoting and supporting breastfeeding.

### 3.3 CHINS characteristics

1. **CHINS value**

A set of questions was asked about how the respondents valued CHINS on a scale of zero to 10 (0 being the least and 10 being the highest value). The following statements were rated:

a. When you use CHINS, how familiar does it feel?
b. Do you feel CHINS is currently a normal part of your work?
c. Staff in your organisation have a shared understanding of the purpose of CHINS.
d. I understand how CHINS affects the nature of my own work.
e. I can see the potential value of CHINS for my work.

A total score was created by adding each of the ratings to get a maximum score of 50. A binary variable was then created (less vs. high value) based on the distribution of the data, whereby a sum of (0–40) was rated as less value, and a score of (41–50) was rated as high value.

2. **CHINS experience**

Respondents were asked to rate a set of 12 statements based on their experience of using CHINS on a scale of zero to five (0 = strongly disagree and 5 = strongly agree). The following statements were rated:

- There are key people who drive the use of CHINS in my organisation.
- I believe that using CHINS is a legitimate part of my role.
- I will continue to use CHINS in my practice.
- I can easily integrate CHINS into my existing work.
- Sufficient training is provided to enable staff to implement CHINS.
- Management adequately supports the use of CHINS.
- Staff agree that CHINS is worthwhile.
- I value the effects CHINS has had on my work.
- I can modify how I work with CHINS.
- I have shared CHINS with other colleagues.
- I have shared CHINS with breastfeeding mothers.
- Breastfeeding mothers agree that CHINS is worthwhile.

For each statement, the data was further reduced to a binary variable based on the distribution of the data (Agree and Strongly agree = 1; Disagree, Strongly disagree and Neither = 0).

3. **Breastfeeding knowledge and education**

Four statements were rated based on satisfaction and confidence around breastfeeding knowledge and education. These statements were rated on a scale of one to five (0 = not at all confident and 5 = very confident) and (0 = highly dissatisfied and 5 = highly satisfied). Binary variables were created based on the distribution of the data. Satisfaction was coded as follows (highly satisfied and satisfied = 1 and highly dissatisfied, dissatisfied and somewhat satisfied = 0). Confidence was coded as follows (very confident and confident = 1 and not at all confident, not confident, and somewhat confident = 0).

The following questions were rated.

a. How satisfied are you with your current level of breastfeeding knowledge and training (0 highly disatisfied and 5 = highly satisfied)
b. How confident do you feel in providing breastfeeding education to peers? (0 = not at all confident and 5 = very confident)
c. How confident do you feel in providing breastfeeding education to students in your field?
d. How confident do you feel in providing breastfeeding education and support to service users?

### 3.4 Statistical analysis

Initially, we determined the distribution of the variables in the data set through histograms, summaries and frequency tables. We combined some categories with low frequencies into one category for categorical variables. Since most continuous variables were skewed, we presented the median and interquartile range (IQR). Furthermore, we created meaningful categories from the continuous variables for analysis. We then conducted bivariate and multivariate analyses to determine the association between the independent variables and each dependent variable. This was done using logistic regression analysis, and we presented odds ratios and confidence intervals in the tables (Supporting Information S1: Table 3a–d). All independent variables that were statistically significant or approached significance at *p* < 0.10 in the bivariate analysis were included in the multivariate analysis. Significance was set at the 95% level for the multivariate analysis (Tables 7a and 7b). The tables for the multivariate analysis only show variables that approached significance from the bivariate analysis.

### 3.5 CHINS characteristics

The following tables show the distribution and descriptive analysis of the variables that were used in the bivariate and multivariate analysis. About 95% of respondents mentioned that they had previously heard of CHINS. The main source of awareness was UNICEF training (61%), university (42%), employer (42%) and colleagues (18%).

### 3.6 CHINS value

Most of the participants highly rated the value of CHINS in their work, as shown in Table 4. Overall, 83% of participants rated CHINS highly (median 47 [out of a total score of 50], IQR [7.5]).

**Table 4:** CHINS value.

	Less value (score 0–5)		More value (score 6–10)	
CHINS value statements	*N*	%	*N*	%
When you use CHINS, how familiar does it feel?	6	5.6	102	94.4
Do you feel CHINS is currently a normal part of your work?	12	11.0	97	89.0
Staff in your organisation have a shared understanding of the purpose of CHINS.	17	15.7	91	84.3
I understand how CHINS affects the nature of my own work.	8	7.4	100	92.6
I can see the potential value of CHINS for my work.	6	5.5	103	94.5
**Overall score** (less value = 0–40; high value = 41–50)^a^	18	16.7	90	83.3

a Median (47), inter‐quartile range (IQR) (7.5), minimum (12), maximum (50).

### 3.7 CHINS experience

Overall, the participants highly rated their experience with CHINS in their work. The statements that had the highest ratings were ease of integrating CHINS into existing work (96%), continuation of the use of CHINS in their practice (95%), the belief that CHINS was a legitimate part of their role (92%), valuing the effects that CHINS had on their work (92%) and sharing CHINS with breastfeeding mothers (90%) (see Table 5).

**Table 5:** CHINS experience.

CHINS experience	Agree		Disagree/neither	
	*N*	%	*N*	%
I can easily integrate CHINS into my existing work	104	96.3	4	3.7
I will continue to use CHINS in my practice	103	94.5	6	5.5
I believe that using CHINS is a legitimate part of my role	100	91.7	9	8.3
I value the effects CHINS has had on my work	99	91.7	9	8.3
I have shared CHINS with breastfeeding mothers	97	89.8	11	10.2
I can modify how I work with CHINS	95	88.0	13	12.0
I have shared CHINS with other colleagues	90	82.6	19	17.4
Sufficient training is provided to enable staff to implement CHINS	87	79.8	22	20.2
There are key people who drive the use of CHINS in my organisation	85	78.0	24	22.0
Staff agree that CHINS is worthwhile	78	73.6	28	26.4
Breastfeeding mothers agree that CHINS is worthwhile	77	72.0	30	28.0
Management adequately supports the use of CHINS	74	69.8	32	30.2

#### 3.7.1 Breastfeeding knowledge and education

The participants generally expressed a high degree of satisfaction with their current levels of breastfeeding knowledge and training (84%). They also felt confident in transferring their breastfeeding knowledge through education to peers (81%), students (82%) and service users (87%) (see Table 6).

**Table 6:** Breastfeeding knowledge and education.

	Satisfied		Not satisfied	
Satisfaction	*N*	%	*N*	%
How satisfied are you with your current level of breastfeeding knowledge and training?	97	84.4	18	15.7

	Confident		Not confident	
Confidence	*N*	%	*N*	%
How confident do you feel in providing breastfeeding education to peers?	93	80.9	22	19.1
How confident do you feel in providing breastfeeding education to students in your field?	93	82.3	20	17.7
How confident do you feel in providing breastfeeding education and support to service users?	99	86.8	15	13.2

### 3.8 Factors associated with confidence in breastfeeding education and CHINS

Socio‐demographic and other key attributes were evaluated for their association with confidence and satisfaction with breastfeeding education and CHINS. Supporting Information S1: Table 3a–d shows the bivariate associations for each of the listed factors. In the bivariate analysis, a few factors were significantly associated with most CHINS attributes, including duration employed as a qualified clinician, duration since the last training was conducted, and factors driving one's work in promoting and supporting breastfeeding. Using the backward elimination method, the final multivariate models only included variables that were significant at a bivariate level. After controlling for all covariates in subsequent models, only a few significant factors remained in the multivariate analysis. We have summarised the multivariate analysis findings in the same sequence in which the column headers (dependent variables) appear in Tables 7a and 7b for ease of reading.

- **Confident in providing breastfeeding education to peers:** Factors that were associated with confidence in providing breastfeeding education to peers included being employed as a qualified clinician for longer than 10 years (OR: 5.09, 95% CI [1.19–21.86] and UNICEF BFI as a training provider (OR 7.13, 95% CI (1.75, 18.97]).
- **Confident in providing breastfeeding education to students:** Factors that were associated with confidence in providing breastfeeding education to students included being employed as a qualified clinician for longer than 10 years (OR: 14.45; 95% CI [2.58–20.02]), UNICEF BFI training provider (OR 9.50; 95% CI [2.01, 23.93]) and borderline significance for having completed the CHINS training more than 12 months after the survey was conducted (OR 3.92, 95% CI [0.84, 18.30]).
- **Confident in providing breastfeeding education to service users:** Factors that were associated with confidence in providing breastfeeding education to service users included being employed as a qualified clinician for longer than 10 years (OR: 10.56, 95% CI [2.60–42.93]), being employed for 6 to 10 years (OR: 5.71, 95% CI [1.04–31.35]), and educating about or promoting and supporting breastfeeding (OR: 3.79, 95% CI [1.06–13.50]).
- **Value of CHINS (high vs. low):** Having received UNICEF BFI training was the only factor that was associated with having a high value of CHINS (OR 5.19, 95% CI [1.05, 25.60]).
- **Key people drive the use of CHINS in my organisation:** Professional duty as a key driver in promoting and supporting breastfeeding had a borderline association with this statement (OR 4.05, 95% CI [0.93, 17.61]).
- **Sufficient training is provided to staff to implement CHINS:** Factors that were associated with sufficient training being provided to staff to implement CHINS were professional duty as a key driver in promoting and supporting breastfeeding (OR 6.50, 95% CI [0.84, 50.34]) and a negative association for having a higher degree (Masters/Doctorate) (OR 0.24, 95% CI [0.07, 0.91]).
- **Management adequately supports the use of CHINS:** Factors associated with management support included working every day in educating and promoting breastfeeding (OR 2.45, 95% CI [1.00, 6.03]), negative borderline significance for having received training from an educational provider (OR 0.42, 95% CI [0.17, 1.04]), and borderline significance for professional duty as a key driver in promoting and supporting breastfeeding (OR 4.46, 95% CI [0.92, 21.71]).
- **I value the effects CHINS has had on my work:** Professional duty as a key driver in promoting and supporting breastfeeding was the only factor significantly associated with valuing the effect that CHINS had on their work (OR 9.40, 95% CI [1.80, 49.06]).
- **I can modify how I work with CHINS:** Factors that were negatively associated were employment in NHS (OR 0.14, 95% CI [0.01, 1.45]) or Local Authority (OR 0.04, 95% CI [0.00, 0.60]), or having completed the training more than 12 months ago since the survey was conducted (OR 0.13, 95% CI [0.01, 1.23]). Working every day to educate and promote breastfeeding was positively associated with this statement: (OR 5.60, 95% CI [1.23, 25.61]).
- **I have shared CHINS with other colleagues:** NHS as a training provider (OR 3.17, 95% CI [1.06, 9.48]) and professional duty as a key driver in promoting and supporting breastfeeding (OR 5.28, 95% CI [1.12, 24.93]) were associated with sharing CHINS with other colleagues.

**Table 7a:** Multivariate analysis of breastfeeding education and CHINS.

			Confident in providing breastfeeding education to peers		Confident in providing breastfeeding education to students		Confident in breastfeeding education and support to service users		Value of CHINS (high vs. low value)		Key people drive the use of CHINS in my organisation	
Characteristic	*N*	%	OR	95% CI	OR	95% CI	OR	95% CI	OR	95% CI	OR	95% CI
**Length as a qualified clinician**												
0–5 years	25	22.7	*Reference*		*Reference*		*Reference*		*Reference*		*Reference*	
6–10 years	25	22.7	1.04	(0.20, 5.48)	1.43	(0.24, 8.62)	5.71*	(1.04, 31.35)	NR	NR	NR	NR
More than 10 years	60	54.6	5.09*	(1.19, 21.86)	14.45**	(2.58, 20.02)	10.56**	(2.60, 42.93)	NR	NR	NR	NR
**Training provider focused on promotion and support for breastfeeding**												
UNICEF BFI	68	60.2	7.13**	(1.75, 18.97)	9.50**	(2.01, 23.93)	NR	NR	5.19*	(1.05, 25.60)	NR	NR
Educational provider	36	31.9	0.33	(0.09, 1.18)	NR	NR	NR	NR	NR	NR	NR	NR
**How long has it been since you completed this training?**												
0–12 months	42	38.5	*Reference*		*Reference*		*Reference*		*Reference*		*Reference*	
More than 12 months	67	61.5	NR	NR	3.92^#^	(0.84, 18.30)	NR	NR	NR	NR	NR	NR
**How often does your role involve educating about and or promotion and support of breastfeeding?**												
Rarely/Weekly	45	39.1	*Reference*		*Reference*		*Reference*		*Reference*		*Reference*	
Daily	70	60.9	NR	NR	NR	NR	3.79*	(1.06, 13.50)	NR	NR	NR	NR
**What drives your work in promoting and supporting breastfeeding**												
Professional duty	105	91.3	2.09	(0.33, 13.22)	NR	NR	NR	NR	NR	NR	4.05#	(0.93, 17.61)
UNICEF accreditation	73	63.5	NR	NR	NR	NR	NR	NR	NR	NR	1.53	(0.60, 3.89)
Employer guidance	43	37.4	NR	NR	NR	NR	NR	NR	NR	NR	2.20	(0.80, 6.11)

*Note*: Logistic regression 95% significance level: ^#^p ≤ 0.10. NR represents p ≤ 0.001; NR, Not reported because the factors were not significant at a bivariate level and, therefore, were not entered into the multivariate analysis.

* p ≤ 0.05;

** p ≤ 0.01.

**Table 7b:** Multivariate analysis of breastfeeding education and CHINS.

			Sufficient training is provided to staff to implement CHINS		Management adequately supports the use of CHINS		I value the effects CHINS has had on my work		I can modify how I work with CHINS		I have shared CHINS with other colleagues	
Characteristic	*N*	%	OR	95% CI	OR	95% CI	OR	95% CI	OR	95% CI	OR	95% CI
**Employer**												
University	29	26.6	*Reference*		*Reference*		*Reference*		*Reference*		*Reference*	
NHS	71	65.1	NR	NR	NR	NR	NR	NR	0.14#	(0.01, 1.45)	NR	NR
Local Authority	9	8.3	NR	NR	NR	NR	NR	NR	0.04*	(0.00, 0.60)	NR	NR
**Qualification**												
Degree	67	61.5	*Reference*		*Reference*		*Reference*		*Reference*		*Reference*	
Diploma	14	12.8	2.39	(0.21, 27.27)	NR	NR	NR	NR	NR	NR	NR	NR
Masters/Doctorate	28	25.7	0.24*	(0.07, 0.91)	NR	NR	NR	NR	NR	NR	NR	NR
**Training provider focused on promotion and support for breastfeeding**												
NHS employer	68	60.2	NR	NR	NR	NR	NR	NR	NR	NR	3.17*	(1.06, 9.48)
UNICEF Train the Trainer	46	40.7	1.98	(0.53, 7.30)	NR	NR	NR	NR	NR	NR	NR	NR
Educational provider	36	31.9	0.53	(0.15, 1.86)	0.42#	(0.17, 1.04)	NR	NR	NR	NR	NR	NR
**How long has it been since you completed this training?**												
0‐12 months	42	38.5	*Reference*		*Reference*		*Reference*		*Reference*		*Reference*	
More than 12 months	67	61.5	NR	NR	NR	NR	NR	NR	0.13#	(0.01, 1.23)	NR	NR
**How often does your role involve educating about and or promoting and supporting breastfeeding?**												
Rarely/Weekly	45	39.1	*Reference*		*Reference*		*Reference*		*Reference*		*Reference*	
Daily	70	60.9	2.03	(0.57, 7.21)	2.45*	(1.00, 6.03)	NR	NR	5.60*	(1.23, 25.61)	NR	NR
**What drives your work in promoting and supporting breastfeeding**												
Professional duty	105	91.3	6.50#	(0.84, 50.34)	4.46#	(0.92, 21.71)	9.40**	(1.80, 49.06)	NR	NR	5.28*	(1.12, 24.93)
UNICEF accreditation	73	63.5	2.26	(0.64, 8.00)	NR	NR	NR	NR	NR	NR	NR	NR

*Note*: Logistic regression 95% significance level:

# p ≤ 0.10. NR represents p ≤ 0.001; NR, Not reported because the factors were not significant at a bivariate level and, therefore, were not entered into the multivariate analysis.

* p ≤ 0.05;

** p ≤ 0.01.

### 3.9 Integrating the data

After the survey and focus group data had been independently analysed, the research team looked for evidence of convergence, divergence and contradiction between the two data sets (Guest & Flemming, 2015). Key findings were then combined using the matrix technique below based on the approach outlined by Plano Clark et al. (2010) and are presented together in Table 8.

**Table 8:** Integrated survey and focus group findings.

1.	2. Survey findings	3. Focus group findings	4. Analysis	5. NPT construct
UNICEF Baby Friendly Initiative training	Improved confidence in providing breastfeeding education to students and peers. If training was received by UNICEF and not other providers, CHINS was more valued.	“I think obviously CHINS… With it being so simple, it's given people the confidence to, kind of, discuss, you know, the principles of positioning with parents”. (R1) “And it [CHINS] is important. And, you know, a lot… You know, they [mothers] need to know what they're looking for, what that good latch is. And staff need to feel confident in that”. (R12) “We use it [CHINS]alongside the fact that UNICEF is really established in wanting to use that […] to push for all of the Trusts within the local region to become UNICEF accredited. But I certainly know that all of the universities in the region are pushing towards UNICEF accreditation so that our students are just ready to hit the… to hit the workforce with this UNICEF‐accredited training already”. (R7)	There is confidence in UNICEF—it is considered a credible source	**Coherence** Factors that drive or inhibit routine implementation of CHINS. **Collective Action** Individual and organisational implementation and promotion of CHINS
Everyday practice providing breastfeeding support/training	Associated with management supporting the use of CHINS and the ability of practitioners to modify how they work with/implement CHINS	“It [CHINS] actually really gives something that's, like, grounded in… in what you're doing”. (R3) “It's simple and easy to teach, as well as… You know…for both the midwives and the mothers”. (R9) “But I use CHINS every day. We, sort of, adopted it… It came through UNICEF, obviously, for us as well, as a Trust, as a board. So, all our staff have UNICEF training, you know, as the requirements”. (R14) “And I think now there's such a push, isn't there, with the NHS Long‐term Plan that all maternity services need to be accredited”. (R15)	Simplicity of CHINS improves the feasibility of implementation for practitioners and their perceptions of its usability for mothers.	**Collective Action** Individual and organisational implementation and promotion of CHINS **Reflexive Monitoring** How is CHINS appraised and modified by staff in organisational settings?
Length/duration of practice	If the length of practice was more than 10 years, the staff were more confident in providing BF education to students, peers, and service users.	“I suppose I would class myself as an expert, but it's just nice with a busy clinic of 31 mums, that you know, I just have to remember CHINS… and I don't have to think oh my God”. (R12) “I've worked as an NHS midwife for a number of years, and my last couple of years in that role, I was working in the local breastfeeding clinics, which ran five days a week. So, I was providing lots of breastfeeding advice and support. […] So, I've been talking to women directly and giving information about understanding what's important in terms of establishing breastfeeding. And, of course, position and attachment and CHINS is very much in there”. (R16) “In the NHS, the turnaround of staff is just so huge and so continuous. So, you depend on, kind of, having regular reminder training sessions, which aren't always facilitated when services are so busy”. (R15)	Sustaining skills/CPD/attrition	**Coherence** Factors that drive or inhibit the routine implementation of CHINS. **Cognitive Participation** How CHINS is promoted/sustained in organisational settings
Professional role and duty	Professional duty in supporting BF was associated with people driving the use of CHINS in the organisation and feeling that sufficient training had been provided to use CHINS and where staff perceived that its use was supported adequately by management. Also associated with the extent to which individual clinician valued effect of CHINS on their work and their likelihood of sharing it with others.	“You've got to empower her to understand what she has to do to get her baby to the breast. So, by giving her that chins, you've done it in a succinct way, and then you've documented that.” (R14) “I'm using it [CHINS] so that they understand… Because it's really important, I think, for midwives to be able to support a mother who's breastfeeding”. (R16) “So, to give them something that can be a tool they can use… That they know everyone else is using as well. And that it isn't personal to them—it's not them saying, oh, I think you should do it like this because… They're just saying, this [CHINS] is the way you should do it, and it's very… It's very professional”. (R10) “But students come out of the unis knowing it […] So, it's things like that as well that keeps it… Keeps it up there as well, when it's not just within practice. It's in education”. (R2)	Practitioners' recognition of BF education and practice as an important part of their professional role.	**Coherence** Factors that drive or inhibit the routine implementation of CHINS. **Cognitive Participation** How CHINS is promoted/sustained in organisational settings **Collective Action** Individual and organisational implementation and promotion of CHINS
The context of providing breastfeeding training.	If you are employed in the NHS and Local Authority, there is a negative association with the ability to modify how CHINS is used. There is a positive association if staff worked in the NHS in relation to sharing CHINS with colleagues.	“We give them out to staff members [small cards with CHINS on] to say that it was, you know, an aide memoir with them, but also so that they could, kind of use that with mams” (R 5) “A couple of years ago, we had the Breastfeeding Network come and do some peer support on the ward, and they didn't use CHINS […], and they ended up using CHINS because we were like, oh, this is what you need” (R11) “We [university training] put an extra S on it. So, we had CHINS‐S. So, sustainable, and then we had safe in there as well”. (R10) So, I think the general crux of the CHIN mnemonic is definitely… We'll [university training] always go back to that. And we probably won't deviate too much from it, to be honest (R7)	Modifying the intervention to match the individual and the context.	**Cognitive Participation** How CHINS is promoted/sustained in organisational settings. **Reflexive Monitoring** How CHINS appraised and modified by staff in organisational settings
Level of education	Less likely to think that you had sufficient training to implement if you have a Masters or Doctorate (academics)	“That's all I've ever used to teach breastfeeding because that's the way I was taught. So, first and foremost, I would teach [CHINS]– if it fits with current evidence. Which… Yeah, there's nothing saying anything has really changed there. So, I would use that. Probably we use it alongside the fact that UNICEF are really established in wanting to use that. And, I mean, that is something, when you go to the study day, that they do. It's in their slides, and they do use the CHIN mnemonic.” (R7—university academic).	Heightened awareness of evidence‐based practice	**Reflexive Monitoring** How is CHINS appraised and modified by staff in organisational settings?

## 4 DISCUSSION

The findings of this first UK mixed methods evaluation of CHINS indicate that CHINS has become a normalised feature of breastfeeding education and practice. Corrigan et al. (2021) suggest that the coherence element of NPT is about “*understanding the WHY*”; the reasons why an innovation in practice is adopted. So, coherence in relation to CHINS refers to the shared understanding of its aims, what the mnemonic means, and how it is used in breastfeeding education and practice (Corrigan et al., 2021). Views of CHINS were overwhelmingly positive, with only one survey respondent stating that they considered it unhelpful and over‐simplistic. In contrast, other respondents found the simplicity of the tool to be part of its value, as it facilitated understanding, retention, and recall of theory for practice **(coherence).** Indeed, one focus group participant made the following comment: *“You've not invented a new drug or a special medicine for making… But it's as effective as that. That's what it is. It's a… As you say, it's a communication tool for breastfeeding” (R13).*

In the context of complex and busy practice settings, some respondents felt CHINS was easy to integrate into their everyday practice and helped them to feel more confident in providing education and support to students and peers, as well as breastfeeding mothers. The survey data indicated that practitioners employed in the NHS and Local Authorities were less likely to feel able to modify CHINS, but this was not reflected in the focus group data, where there were examples of adding to the memory aide (R10) and producing materials such as flashcards for showing and sharing with peers and breastfeeding mothers. One interesting request by several focus group respondents was for a complementary memory aide for attachment to be developed, given that positioning and attachment are key to successful breastfeeding.

Survey findings show that those who had completed UNICEF BFI training were more likely to value CHINS, and there was also an association between a sense of professional duty to provide high‐quality breastfeeding promotion and support and the perceived value of CHINS. These findings are perhaps explained by the fact that UNICEF adopted CHINS in 2010 and has since continued to use it within their training, as well as the fact that UNICEF BFI training has become the main, evidence‐based approach to training key staff and accrediting services with a key role in promoting and supporting breastfeeding in the United Kingdom, as noted in the NHS Long Term Plan (NHS, 2019). While one focus group participant (R14) noted that if CHINS was not worthwhile, it would not be used, regardless of UNICEF's role in embedding it across the United Kingdom, several reported feeling reassured that if UNICEF were using it, this meant it was relevant, and an evidence‐based approach to high‐quality practice. Equally, several respondents were documenting CHINS in their clinical records as evidence they had given evidence‐based, high‐quality care, and they were reassured that good practice had been delivered when they saw it in the records of their colleagues. Given the regulatory and legislative mandate for all nurses and midwives to keep clear and accurate records (Council, 2018; Royal College of Nursing, 2023), this provides some evidence of the acceptability of healthcare professionals to use CHINS in professional practice.

While there was a correlation between the length of service/duration of time in practice and increased confidence in using CHINS, it was interesting that survey respondents with a Master's or Doctoral Level qualification were less likely to be satisfied with their level of breastfeeding education. This might be explained by the fact that practitioners with higher academic qualifications are more likely to work in academic and clinical leadership roles (Rees et al., 2019), where there is less emphasis on regular breastfeeding training. This would warrant further exploration and perhaps suggests a training need, given their important role in leading services and educating the future breastfeeding workforce. This finding was not reflected in the focus group data, but there was generally a heightened awareness of the need for evidence‐based practice. Duration of service and experience and increased confidence in delivering breastfeeding education and practice are important considerations concerning how training is targeted and how less experienced staff can be supported to gain confidence in this important area of practice.

Cognitive participation refers to the process and work that individuals do to engage others in using the intervention, what Corrigan et al. (2021) refer to as “*generating the will*”. As outlined above, the strategic policy drivers for using CHINS were evident, but staff were driving forward the use of CHINS in their own practice and in the practice of others. Indeed, participants referred to how CHINS had helped structure and standardise their approach to giving advice about correct positioning (R12). A recent scoping review by Beggs et al. (2021) reported that breastfeeding mothers want sound and practical advice from healthcare providers, particularly about latching on and dealing with issues such as pain, but unfortunately, some felt the support and information they received was inadequate, impractical, or infused with conflicting messaging. Mixed messages can lead to mistrust of healthcare providers (Carpenter et al., 2016) but also have a differential impact on some mothers, particularly those with lower levels of health literacy (Crondahl & Eklund Karlsson, 2016) or wider support to help with breastfeeding (Beggs et al., 2021). It is here that interventions such as CHINS can help address these issues.

While this evaluation found practitioners had recommended CHINS to other professionals and training providers, such as the Breastfeeding Network **(cognitive participation)**, wider dissemination might help, given the consistency participants felt it had produced alongside its transformational impact in helping practitioners move away from outdated advice such as “tummy to mummy” (R1), which is no longer recommended. Equally, wider dissemination may address some of the challenges noted in practice, including lack of enthusiasm (R2) and the absence of good support (R8) for breastfeeding.

Collective action refers to how the intervention is enacted. High levels of collective action were apparent in this evaluation, whereby CHINS had made some areas of practice easier and, as such, CHINS was easily incorporated into everyday practice (Huddlestone et al., 2020). While there were high levels of strategic commitment for all key staff to complete UNICEF BFI training **(collective action),** survey findings indicate this was more apparent in areas where staff had a daily responsibility to provide breastfeeding promotion and support. Focus group findings suggest this generally appeared difficult to achieve. Reasons cited for this included staff attrition and shortages. It is widely acknowledged that staffing is a key concern across health and social care, with an estimated shortfall of 30,000 nurses (The Kings Fund, 2022), (2550 midwives (Midwives, 2022) and 5000 health visitors (Institute for Health Visiting, 2023). While continuing professional development (CPD) is a fundamental professional requirement, it is known that CPD is often not prioritised in the presence of workforce pressures, meaning some staff may not have the requisite skills to promote and support breastfeeding effectively. UNICEF BFI training is considered highly effective but requires significant investment and staff time to attend. Usually, training is a minimum of 2 days, which has implications in areas with high staff turnover or those experiencing staffing shortages. Given the impact and simplicity of CHINS, it might be suggested that a more condensed approach to training, focusing on key principles such as positioning and attachment, could help ensure all have key skills in their absence and complement more in‐depth training when possible. Indeed, shortened CPD approaches are considered a valuable way to develop and maintain skills, particularly given staffing shortages (Royal College of Nursing, 2019b). Shortened training and some examples of developing CHINS further in the form of posters and infographics, as recommended by participants, may also facilitate its wider roll‐out for other staff groups, including GPs, who were identified as an essential group in this evaluation **(reflexive monitoring).**

### 4.1 Strengths and limitations

The mixed methods approach is a key strength of this first evaluation of the memory aide CHINS, providing a more comprehensive approach to answering the research question. However, it must be noted that the survey response rate was lower than the target sample size, which is a key limitation of the evaluation. It was positive that there was representation from practitioners working across the United Kingdom in various roles. Still, it must be noted that the survey sample was dominated by respondents from a midwifery and academic background, which is also reflected in the focus group sample. Therefore, it is noted that the sample was female and mainly from a White background, which is, to some extent, reflective of the lack of diversity in the workforce generally. A larger sample would have provided an opportunity for a more diverse sample and enhanced the significance of the results.

### 4.2 Recommendations

This evaluation provides evidence that CHINS has a role in supporting the education, training, and practice of the breastfeeding workforce. However, it also highlights the need for the memory aide to be disseminated more widely, and consideration should be given to how it can be adapted for use in different settings. The results suggest a need to explore in more detail the link between practitioners with higher academic qualifications and their confidence in providing breastfeeding education and support, as well as how to ensure staff build and maintain confidence and skills in the presence of workforce challenges. A potential area for exploration is the role of condensed approaches to providing breastfeeding education and training, how simple interventions such as CHINS could add value, and the development of a complementary memory aide for the principles of attachment for effective breastfeeding.

## 5 CONCLUSION

It is clear from this mixed methods evaluation that CHINS has become a well‐established feature of breastfeeding education and practice across the United Kingdom. This appears to be largely due to its simplicity, which makes it easy to remember, recall and use in everyday practice and more so because UNICEF has adopted it. This research provides some evidence to support its use in clinical practice and its role in bringing about a standard approach to promoting, supporting, and assessing principles of positioning for effective breastfeeding. Based on the findings of this evaluation, there are clear advantages to sharing CHINS more widely in terms of its extensive inclusion in educational materials and within breastfeeding education and training beyond the United Kingdom. Future research should focus on understanding the utility of CHINS in wider settings across and beyond the United Kingdom and how it can be further adapted to support breastfeeding education and practice.

## EVALUATION ACTIVITIES AND FINDINGS

- Methods—A UK survey was completed by 115 practitioners and academics.
- 16 of the survey respondents took part in focus groups.
- Findings show that CHINS is widely used. It is simple and easy and has introduced a standardised approach to the principles of positioning for effective breastfeeding.

## AUTHOR CONTRIBUTIONS

Lynette Shotton conducted data collection. Lynette Shotton and Tracy Collins analysed the qualitative data and identified themes from the data. Reinie Cordier and Fadzai Chikwava analysed the quantitative data. Lynette Shotton, Tracy Collins, Reinie Cordier developed the draft and this was read, developed and approved by all authors.

## CONFLICT OF INTEREST STATEMENT

The authors declare no conflict of interest.

## Supporting information

Supporting information.

## Data Availability

The data sets generated and/or analysed during the current study are not publicly available but are available from the corresponding author on reasonable request.
