# Supplementary material for: A mixed methods evaluation of the breastfeeding memory aide CHINS
Source: Matern Child Nutr. 2024 Jul 19;20(4):e13704. doi: 10.1111/mcn.13704 (PMC11574650; doi:10.1111/mcn.13704)
Supplement: Supplementary file 1 — Supporting information. [file MCN-20-e13704-s001.docx]

**Supplementary File 1. Online survey**

| Question |  | NPT Construct |
| --- | --- | --- |
| 1 What is your professional background | Midwife, health visitor, university educator, college educator, UNICEF BFI educator – other (please specify) |  |
| 2. Who is your employer | NHS, LA; University, College; private sector, UNICEF – other -please specify |  |
| 3. How long have you worked for this organisation? | 0-1; 1-2; 3-5; 6-10; 11-15; more than 15 years |  |
| 4. How long have you been qualified? | 0-1; 1-2; 3-5; 6-10; 11-15; more than 15 years |  |
| 5. What is your highest level of professional qualification | Diploma, degree, Masters, Doctoral other-please specify |  |
| 6. What is your gender? |  |  |
| 7. What is your ethnic group |  |  |
| 8. How old are you? | 21-30; 31-45; 46-55; 55-67; older than 67 |  |
| 9. Have you had specific training about breastfeeding | Yes / No (No -go to Q 13) |  |
| 10. Was this training: | UNICEF 2 day; UNICEF Train the Trainer, Other – please specify |  |
| 11. Who provided / organized this training? | (Employer, self, other – please specify |  |
| 12. Was this training in the last: | 12 months; 1-2 years; 2-3 years; longer -please specify |  |
| 13. Do you feel breastfeeding education is relevant to your role? | Agree, not sure, disagree |  |
| 14. Would you like breastfeeding training? | Yes / No |  |
| 15. Who should provide this? | Employer; UNICEF, other – please specify |  |
| 16. How often? | Annual, bi-annual, other – please specify |  |
| 17. How often do you engage in CPD focused on breastfeeding? | Annually, bi-annually; rarely; never  (Option 1 and 2 go to next Q; Other to Q19 |  |
| 18. Is this CPD delivered by: | Employer; UNICEF; private provider; self-directed; other – please specify |  |
| 19. Does your work involve promotion of breastfeeding: | Daily; weekly; monthly; rarely; never |  |
| 20. Can you briefly describe your work in this area | Free text |  |
| 21. Does your work involve providing breastfeeding education: | Daily, weekly, monthly; rarely; never |  |
| 22. Can you briefly describe your work in this area | Free text |  |
| 23.Does your work involve providing advice to: | Breastfeeding mothers; colleagues; students training to be midwives / health visitors. |  |
| 24. Can you briefly describe your work in this area. | Free text |  |
| 25. How confident do you feel in providing breastfeeding education to: | Peers; student midwives / health visitors; breastfeeding mothers (scale confident, somewhat confident, a little confident, not at al) |  |
| 26. What drives your work in supporting breastfeeding promotion/ practice | Professional interest; UNICEF accreditation; employer; professional regulatory body |  |
| 27. Are there any barriers to your effectiveness in promotion and support of breastfeeding? | Free text |  |
| Thinking about the memory aide CHINS | |  |
| 28. Have you heard of CHINS | Yes /No (No go to X) |  |
| 29. Where did you learn about CHINS? | UNICEF; CPD; employer; university / college, other -please specify |  |
| 30. How often do you use CHINS? | Daily; weekly; monthly; rarely; never |  |
| 31. Do you find CHINS helpful? | Definitely agree; agree; disagree | Reflexive monitoring |
| 32. When you use CHINS how familiar does it feel? | Scale of 0-10 | Coherence |
| 33. Do you feel CHINS is currently a normal part of your work? | Scale of 0-10 | Coherence |
| 34. Staff in your organization have a shared understanding of the purpose of CHINS? | Scale of 0-10 | Coherence |
| 35. I understand how CHINS affects the nature of my own work | Scale of 0-10 | Cognitive participation |
| 36. I can see the potential value of CHINS for my work | Scale of 0-10 | Coherence |
| 37. There are key people who drive the use of CHINS in my organization | Strongly Agree-Disagree | Collective action |
| 38. I believe that using CHINS is a legitimate part of my role | Strongly Agree-Disagree | Cognitive participation |
| 39. I will continue to use CHINS in my practice | Strongly Agree-Disagree | Cognitive Participation |
| 40. I can easily integrate CHINS into my existing work | Strongly Agree-Disagree | Collective action |
| 41. Sufficient training is provided to enable staff to implement CHINS | Strongly Agree-Disagree | Collective action |
| 42. Management adequately support the use of CHINS | Strongly Agree-Disagree | Collective action |
| 43. Staff agree that CHINS is worthwhile | Strongly Agree-Disagree | Reflexive monitoring |
| 44. I value the effects CHINS has had on my work | Strongly Agree-Disagree | Reflexive monitoring |
| 45. I can modify how I work with CHINS | Strongly Agree-Disagree | Reflexive monitoring |
| 46. I have shared CHINS with my colleagues | Strongly Agree-Disagree | Cognitive participation |
| 47.I have shared CHINS with breastfeeding mothers | Strongly Agree-Disagree | Cognitive participation |
| 48. |  |  |
| X from Q 28 Those who have not heard of CHINS. Description of CHINS given | |  |
| Do you think you would use CHINS | Yes / No – free text justification |  |
| How might others in your profession use it? | Free text |  |
| How might it be used with breastfeeding mothers? | Free text |  |

**Supplementary File 2. Focus group questions informed by Normalisation Process Theory**

| Focus group interview guide | NPT Construct |
| --- | --- |
| 1. Tell me what you know about the memory aide CHINS | Coherence |
| 1. How do you use CHINS? | Coherence  Cognitive Participation |
| 1. Who do you use CHINS with and why? | Cognitive Participation |
| 1. Has CHINS helped your practice in any way? | Collective Action |
| 1. Has it changed your practice in any way? | Collective Action |
| 1. Are there any limitations to CHINS? | Reflexive Monitoring |
| 1. Have you enhanced it or changed it in any way? | Reflexive Monitoring |
| 1. How could CHINS be developed or improved? | Reflexive Monitoring |
| 1. Is there anything else you would like to add? |  |

**Supplementary File 3a. Table of bivariate analysis of breastfeeding education and CHINS**

| **Characteristic** | |  |  | **Satisfied with current level of breastfeeding knowledge and training** | | **Confident in providing breastfeeding education to peers** | | **Confident in providing breastfeeding education to students** | | **Confident in breastfeeding education and support to service users** | | **Chins value (high vs less value)** | |
| --- | --- | --- | --- | --- | --- | --- | --- | --- | --- | --- | --- | --- | --- |
|  |  | **N** | **%** | **OR** | **95%CI** | **OR** | **95%CI** | **OR** | **95%CI** | **OR** | **95%CI** | **OR** | **95%CI** |
| **Profession** | |  |  |  |  |  |  |  |  |  |  |  |  |
|  | Academic | 33 | 31.3 | *Reference* | |  |  |  |  |  |  |  |  |
|  | Midwife | 45 | 42.4 | 0.83 | (0.24, 2.80) | 0.69 | (0.23, 2.10) | 1.00 | (0.31, 3.22) | 1.50 | (0.44, 5.16) | 1.21 | (0.32, 4.62) |
|  | Health visitor | 28 | 26.4 | 2.32 | (0.41, 13.02) | 2.89 | (0.53, 15.63) | 2.89 | (0.53, 15.63) | 6.23# | (0.70, 55.36) | 1.03 | (0.25, 4.27) |
| **Employer** | |  |  |  |  |  |  |  |  |  |  |  |  |
|  | University | 29 | 26.6 | *Reference* | |  |  |  |  |  |  |  |  |
|  | NHS | 71 | 65.1 | 0.87 | (0.25, 3.00) | 1.42 | (0.47, 4.30) | 1.42 | (0.47, 4.30) | 1.99 | (0.57, 6.89) | 0.88 | (0.25, 3.08) |
|  | Local Authority | 9 | 8.3 | 1.28 | (0.12, 13.17) | 0.91 | (0.15, 5.58) | 0.87 | (0.18, 4.19) | 0.87 | (0.18, 4.27) | 0.40 | (0.08, 1.92) |
| **Length with organisation** | | | | | | | | | |  |  |  |  |
|  | 0-5 years | 50 | 43.5 | *Reference* | |  |  |  |  |  |  |  |  |
|  | 6+ years | 65 | 56.5 | 1.37 | (0.50, 3.74) | 2.19 | (0.85, 5.63) | 2.38# | (0.89, 6.37) | 2.12 | (0.70, 6.42) | 1.20 | (0.43, 3.29) |
| **Length as qualified clinician** | | | | | | | | | |  |  |  |  |
|  | 0-5 years | 25 | 22.7 | *Reference* | |  |  |  |  |  |  |  |  |
|  | 6-10 years | 25 | 22.7 | 5.41# | (1.02, 28.79) | 3.70* | (1.05, 12.96) | 7.00** | (1.64, 29.85) | 6.47* | (1.23, 34.01) | 1.94 | (0.49, 7.76) |
|  | More than 10 years | 60 | 54.6 | 3.06# | (1.00, 9.40) | 10.15*** | (3.04, 33.91) | 11.00*** | (3.26, 37.11) | 7.73** | (2.10, 28.46) | 3.18# | (0.94, 10.72) |
| **Qualification** | |  |  |  |  |  |  |  |  |  |  |  |  |
|  | Degree | 67 | 61.5 | *Reference* | |  |  |  |  |  |  |  |  |
|  | Diploma | 14 | 12.8 | Omitted | | Omitted | | 3.50 | (0.42, 29.10) | Omitted | | 1.18 | (0.23, 6.08) |
|  | Masters/Doctorate | 28 | 25.7 | 1.44 | (0.43, 4.89) | 1.88 | (0.57, 6.24) | 2.15 | (0.57, 8.20) | 1.40 | (0.35, 5.55) | 0.86 | (0.26, 2.82) |
| **Training provider focused on promotion and support for breastfeeding** | | | | | | | | | | | |  |  |
|  | NHS employer | 68 | 60.2 | 1.42 | (0.50, 4.00) | 3.05* | (1.14, 8.12) | 2.58# | (0.94, 7.06) | 3.24* | (1.01, 10.43) | 0.75 | (0.25, 2.20) |
|  | UNICEF Train the Trainer | 46 | 40.7 | 1.79 | (0.58, 5.48) | 19.15** | (2.47, 28.68) | 17.23** | (2.21, 134.51) | 4.69# | (1.00, 22.08) | 4.08* | (1.10, 15.19) |
|  | UNICEF BFI | 68 | 60.2 | 3.34* | (1.14, 9.84) | 9.71*** | (3.00, 31.50) | 6.08** | (2.00, 18.49) | 4.71* | (1.37, 16.13) | 1.61 | (0.57, 4.58) |
|  | Educational provider | 36 | 31.9 | 0.26* | (0.09, 0.75) | 0.16*** | (0.06, 0.44) | 0.34* | (0.12, 0.92) | 0.30* | (0.10, 0.94) | 0.38# | (0.13, 1.09) |
| **How long is it since you completed this training?** | | | | | | | | | |  |  |  |  |
|  | 0-12 months | 42 | 38.5 | *Reference* | |  |  |  |  |  |  |  |  |
|  | More than 12 months | 67 | 61.5 | 4.79* | (1.22, 18.80) | 1.82 | (0.65, 5.06) | 4.66* | (1.37, 15.83) | 4.09# | (1.03, 16.29) | 0.79 | (0.25, 2.52) |
| **How often do you engage in continuing professional development activity focused on breastfeeding?** | | | | | | | | | |  |  |  |  |
|  | Annually | 85 | 80.2 | *Reference* | |  |  |  |  |  |  |  |  |
|  | Bi-annually | 21 | 19.8 | 1.56 | (0.32, 7.58) | 1.18 | (0.31, 4.56) | 4.06 | (0.50, 32.77) | 2.25 | (0.27, 18.86) | 1.20 | (0.31, 4.67) |
| **How often does your role involve educating about and or promotion and support of breastfeeding?** | | | | | | | | | |  |  |  |  |
|  | Rarely/ Weekly | 45 | 39.1 | *Reference* | |  |  |  |  |  |  |  |  |
|  | Daily | 70 | 60.9 | 1.30 | (0.47, 3.58) | 1.38 | (0.54, 3.53) | 2.12 | (0.80, 5.63) | 2.74# | (0.90, 8.34) | 1.29# | (0.37, 4.49) |
| **What drives your work in promoting and supporting breastfeeding** | | | | | | | | | |  |  |  |  |
|  | Professional duty | 105 | 91.3 | 2.57 | (0.60, 11.06) | 3.22# | (0.82, 12.59) | 2.56 | (0.58, 11.24) | 3.29 | (0.75, 14.44) | 1.75 | (0.32, 9.46) |
|  | UNICEF accreditation | 73 | 63.5 | 0.85 | (0.29, 2.45) | 2.00 | (0.78, 5.12) | 2.00 | (0.75, 5.31) | 1.17 | (0.38, 3.54) | 2.33 | (0.83, 6.52) |
|  | Employer guidance | 43 | 37.4 | 0.71 | (0.25, 1.95) | 0.66 | (0.26, 1.69) | 0.52 | (0.20, 1.39) | 0.65 | (0.22, 1.95) | 0.76 | (0.27, 2.11) |

*Notes.* Logistic Regression significance level: #p≤0.10; *p≤0.05; **p≤0.01; ***p≤0.001

**Supplementary File 3b. Table of bivariate analysis of breastfeeding education and CHINS**

| **Characteristic** | |  |  | **Key people drive the use of CHINS in my organisation** | | **I will continue to use CHINS in my practice** | | **CHINS is a legitimate part of my role** | | **I can easily integrate CHINS into my existing work** | | **Sufficient training is provided to staff to implement CHINS** | |
| --- | --- | --- | --- | --- | --- | --- | --- | --- | --- | --- | --- | --- | --- |
|  |  | **N** | **%** | **OR** | **95%CI** | **OR** | **95%CI** | **OR** | **95%CI** | **OR** | **95%CI** | **OR** | **95%CI** |
| **Profession** | |  |  |  |  |  |  |  |  |  |  |  |  |
|  | Academic | 33 | 31.3 | *Reference* | |  |  |  |  |  |  |  |  |
|  | Midwife | 45 | 42.4 | 1.22 | (0.35, 4.23) | 1.19 | (0.07, 19.75) | 0.77 | (0.12, 4.94) | Omitted | | 2.96 | (0.89, 9.79) |
|  | Health visitor | 28 | 26.4 | 0.47 | (0.14, 1.54) | 0.19 | (0.02, 1.79) | 0.39 | (0.07, 2.29) | 0.39 | (0.03, 4.56) | 2.61 | (0.72, 9.50) |
| **Employer** | |  |  |  |  |  |  |  |  |  |  |  |  |
|  | University | 29 | 26.6 | *Reference* | |  |  |  |  |  |  |  |  |
|  | NHS | 71 | 65.1 | 0.69 | (0.23, 2.13) | 0.54 | (0.06, 5.10) | 0.30 | (0.03, 2.52) | 0.74 | (0.07, 7.41) | 2.48 | (0.88, 6.97) |
|  | Local Authority | 9 | 8.3 | 0.57 | (0.13, 2.56) | 0.50 | (0.03, 8.60) | 0.50 | (0.03, 8.60) | Omitted | | 2.22 | (1.01, 4.88) |
| **Length with organisation** | | | |  |  |  |  |  |  |  |  |  |  |
|  | 0-5 years | 50 | 43.5 | *Reference* | |  |  |  |  |  |  |  |  |
|  | 6+ years | 65 | 56.5 | 1.91 | (0.76, 4.77) | 0.57 | (0.10, 3.27) | 0.13# | (0.02, 1.08) | 0.37 | (0.04, 3.72) | 1.95 | (0.75, 5.05) |
| **Length as qualified clinician** | | | |  |  |  |  |  |  |  |  |  |  |
|  | 0-5 years | 25 | 22.7 | *Reference* | |  |  |  |  |  |  |  |  |
|  | 6-10 years | 25 | 22.7 | 2.35 | (0.60, 9.20) | Omitted | | 0.96 | (0.06, 16.24) | No convergence | | 1.25 | (0.29, 5.35) |
|  | More than 10 years | 60 | 54.6 | 1.88 | (0.65, 5.48) | 0.53 | (0.06, 5.01) | 0.34 | (0.04, 2.99) | No convergence | | 1.00 | (0.31, 3.26) |
| **Qualification** | |  |  |  |  |  |  |  |  |  |  |  |  |
|  | Degree | 67 | 61.5 | *Reference* | |  |  |  |  |  |  |  |  |
|  | Diploma | 14 | 12.8 | 1.07 | (0.26, 4.37) | 0.43 | (0.04, 5.14) | 0.90 | (0.09, 8.70) | Omitted | | 1.93 | (0.22, 16.79) |
|  | Masters/Doctorate | 28 | 25.7 | 0.83 | (0.29, 2.37) | 0.27 | (0.04, 1.70) | 0.40 | (0.09, 1.72) | 0.42 | (0.06, 3.18) | 0.22 | (0.07, 0.63) |
| **Training provider focused on promotion and support for breastfeeding** | | | | | | | |  |  |  |  |  |  |
|  | NHS employer | 68 | 60.2 | 1.24 | (0.50, 3.10) | 2.18 | (0.35, 13.61) | 1.13 | (0.29, 4.47) | 1.45 | (0.20, 10.72) | 1.71 | (0.66, 4.47) |
|  | UNICEF Train the Trainer | 46 | 40.7 | 1.31 | (0.52, 3.33) | 0.48 | (0.08, 2.98) | 0.92 | (0.23, 3.64) | 0.23 | (0.02, 2.28) | 2.85 | (0.96, 8.47) |
|  | UNICEF BFI | 68 | 60.2 | 0.81 | (0.31, 2.11) | 0.41 | (0.04, 3.80) | 0.84 | (0.20, 3.55) | 0.55 | (0.05, 5.44) | 2.20 | (0.84, 5.78) |
|  | Educational provider | 36 | 31.9 | 0.63 | (0.25, 1.60) | 0.74 | (0.12, 4.63) | 0.60 | (0.15, 2.37) | 0.16 | (0.02, 1.57) | 0.37 | (0.14, 0.97) |
| **How long has it been since you completed this training?** | | | | | | | |  |  |  |  |  |  |
|  | 0-12 months | 42 | 38.5 | *Reference* | |  |  |  |  |  |  |  |  |
|  | More than 12 months | 67 | 61.5 | 0.85 | (0.30, 2.41) | No convergence | | 0.11# | (0.01, 1.08) | No convergence | | 1.52 | (0.58, 4.00) |
| **How often do you engage in continuing professional development activity focused on breastfeeding?** | | | | | | | | |  |  |  |  |  |
|  | Annually | 85 | 80.2 | *Reference* | |  |  |  |  |  |  |  |  |
|  | Bi-annually | 21 | 19.8 | 2.95 | (0.63, 13.84) | 0.22# | (0.04, 1.17) | 0.45 | (0.10, 2.00) | 0.23 | (0.03, 1.75) | 0.45 | (0.14, 1.37) |
| **How often does your role involve educating about and or promoting and supporting breastfeeding?** | | | | | | | | |  |  |  |  |  |
|  | Rarely/ Weekly | 45 | 39.1 | *Reference* | |  |  |  |  |  |  |  |  |
|  | Daily | 70 | 60.9 | 0.84 | (0.16, 4.54) | 0.73 | (0.12, 4.57) | 0.63 | (0.13, 3.02) | 1.60 | (0.22, 11.82) | 2.89 | (1.11, 7.54) |
| **What drives your work in promoting and supporting breastfeeding** | | | | | | | |  |  |  |  |  |  |
|  | Professional duty | 105 | 91.3 | 4.05# | (0.93, 17.61) | 2.74 | (0.28, 26.82) | 4.48# | (0.76, 26.41) | No convergence | | 8.24 | (1.80, 37.78) |
|  | UNICEF accreditation | 73 | 63.5 | 1.53 | (0.60, 3.89) | 4.24 | (0.74, 24.34) | 1.62 | (0.41, 6.45) | No convergence | | 2.98 | (1.14, 7.77) |
|  | Employer guidance | 43 | 37.4 | 2.20 | (0.80, 6.11) | 3.31 | (0.37, 29.34) | 2.33 | (0.46, 11.81) | No convergence | | 1.44 | (0.53, 3.90) |

*Notes.* Logistic Regression significance level: #p≤0.10; *p≤0.05; **p≤0.01; ***p≤0.001

**Supplementary File 3c. Table of bivariate analysis of breastfeeding education and CHINS**

| **Characteristic** | |  |  | **Management adequately supports the use of CHINS** | | **Staff agree that CHINS is worthwhile** | | **I value the effects CHINS has had on my work** | | **I can modify how I work with CHINS** | |
| --- | --- | --- | --- | --- | --- | --- | --- | --- | --- | --- | --- |
|  |  | **N** | **%** | **OR** | **95%CI** | **OR** | **95%CI** | **OR** | **95%CI** | **OR** | **95%CI** |
| **Profession** | |  |  |  |  |  |  |  |  |  |  |
|  | Academic | 33 | 31.3 | *Reference* |  |  |  |  |  |  |  |
|  | Midwife | 45 | 42.4 | 1.60 | (0.57, 4.46) | 0.85 | (0.29, 2.45) | 1.20 | (0.23, 6.39) | 0.77 | (0.12, 4.94) |
|  | Health visitor | 28 | 26.4 | 1.57 | (0.51, 4.88) | 1.83 | (0.48, 6.95) | 0.80 | (0.15, 4.33) | 0.23# | (0.04, 1.23) |
| **Employer** | |  |  |  |  |  |  |  |  |  |  |
|  | University | 29 | 26.6 | *Reference* |  |  |  |  |  |  |  |
|  | NHS | 71 | 65.1 | 1.94 | (0.75, 5.00) | 1.20 | (0.44, 3.25) | 0.73 | (0.14, 3.85) | 0.61 | (0.12, 3.15) |
|  | Local Authority | 9 | 8.3 | 1.72 | (0.43, 6.90) | 3.20 | (0.34, 29.90) | 0.59 | (0.05, 7.42) | 0.15# | (0.02, 1.09) |
|  |  |  |  |  |  |  |  |  |  |  |  |
| **Length with organisation** | | | |  |  |  |  |  |  |  |  |
|  | 0-5 years | 50 | 43.5 | *Reference* |  |  |  |  |  |  |  |
|  | 6+ years | 65 | 56.5 | 2.03 | (0.56, 7.31) | 1.82 | (0.76, 4.35) | 0.55 | (0.13, 2.34) | 0.99 | (0.31, 3.18) |
| **Length as qualified clinician** | | | |  |  |  |  |  |  |  |  |
|  | 0-5 years | 25 | 22.7 | *Reference* |  |  |  |  |  |  |  |
|  | 6-10 years | 25 | 22.7 | 2.03 | (0.56, 7.31) | 0.60 | (0.18, 2.03) | 0.46 | (0.04, 5.41) | 3.14 | (0.30, 32.48) |
|  | More than 10 years | 60 | 54.6 | 1.22 | (0.45, 3.32) | 1.52 | (0.51, 4.55) | 0.42 | (0.05, 3.77) | 0.70 | (0.17, 2.83) |
| **Qualification** | |  |  |  |  |  |  |  |  |  |  |
|  | Degree | 67 | 61.5 | *Reference* |  |  |  |  |  |  |  |
|  | Diploma | 14 | 12.8 | 2.05 | (0.41, 10.21) | 0.63 | (0.17, 2.34) | 0.91 | (0.09, 8.85) | 1.69 | (0.19, 14.92) |
|  | Masters/Doctorate | 28 | 25.7 | 0.37* | (0.14, 0.95) | 0.36# | (0.13, 0.98) | 0.40 | (0.09, 1.75) | 0.57 | (0.16, 1.99) |
| **Training provider focused on promotion and support for breastfeeding** | | | | | | | | | |  |  |
|  | NHS employer | 68 | 60.2 | 1.54 | (0.66, 3.59) | 1.41 | (0.58, 3.40) | 3.16 | (0.75, 13.39) | 2.19 | (0.65, 7.43) |
|  | UNICEF Train the Trainer | 46 | 40.7 | 1.69 | (0.70, 4.08) | 2.04 | (0.80, 5.20) | 1.50 | (0.35, 6.35) | 0.48 | (0.14, 1.61) |
|  | UNICEF BFI | 68 | 60.2 | 1.33 | (0.57, 3.15) | 1.16 | (0.47, 2.84) | 0.82 | (0.19, 3.50) | 0.52 | (0.13, 2.06) |
|  | Educational provider | 36 | 31.9 | 0.40* | (0.17, 0.94) | 0.52 | (0.21, 1.29) | 0.37 | (0.09, 1.47) | 0.46 | (0.14, 1.55) |
| **How long is it since you completed this training?** | | | |  |  |  |  |  |  |  |  |
|  | 0-12 months | 42 | 38.5 | *Reference* |  |  |  |  |  |  |  |
|  | More than 12 months | 67 | 61.5 | 0.95 | (0.40, 2.25) | 1.24 | (0.51, 3.03) | 0.90 | (0.20, 3.99) | 0.27# | (0.06, 1.29) |
| **How often do you engage in continuing professional development activity focused on breastfeeding?** | | | | | | | | | | | |
|  | Annually | 85 | 80.2 | *Reference* |  |  |  |  |  |  |  |
|  | Bi-annually | 21 | 19.8 | 0.98 | (0.33, 2.85) | 0.60 | (0.21, 1.72) | 0.38 | (0.08, 1.74) | 0.72 | (0.18, 2.94) |
| **How often does your role involve educating about and or promotion and support of breastfeeding?** | | | | | | | | | | | |
|  | Rarely/ Weekly | 45 | 39.1 | *Reference* |  |  |  |  |  |  |  |
|  | Daily | 70 | 60.9 | 2.51* | (1.07, 5.89) | 1.27 | (0.53, 3.05) | 1.28 | (0.32, 5.08) | 2.87# | (0.87, 9.47) |
| **What drives your work in promoting and supporting breastfeeding** | | | | | | | | | | | |
|  | Professional duty | 105 | 91.3 | 4.39* | (0.98, 19.61) | 3.08 | (0.72, 13.28) | 9.40** | (1.80, 49.06) | 1.05 | (0.12, 9.27) |
|  | UNICEF accreditation | 73 | 63.5 | 1.96 | (0.83, 4.65) | 1.46 | (0.59, 3.57) | 1.00 | (0.24, 4.25) | 1.86 | (0.57, 6.00) |
|  | Employer guidance | 43 | 37.4 | 1.38 | (0.58, 3.26) | 1.55 | (0.62, 3.85) | 2.37 | (0.47, 12.01) | 2.32 | (0.60, 8.99) |

*Notes.* Logistic Regression significance level: #p≤0.10; *p≤0.05; **p≤0.01; ***p≤0.001

**Supplementary File 3d. Table of bivariate analysis of breastfeeding education and CHINS**

| **Characteristic** | |  |  | **I have shared CHINS with other colleagues** | | **I have shared CHINS with breastfeeding mothers** | | **Breastfeeding mothers agree that CHINS is worthwhile** | |
| --- | --- | --- | --- | --- | --- | --- | --- | --- | --- |
|  |  | **N** | **%** | **OR** | **95%CI** | **OR** | **95%CI** | **OR** | **95%CI** |
| **Profession** | |  |  |  |  |  |  |  |  |
|  | Academic | 33 | 31.3 | *Reference* | |  |  |  |  |
|  | Midwife | 45 | 42.4 | 0.94 | (0.23, 3.82) | 0.70 | (0.15, 3.20) | 1.01 | (0.34, 2.97) |
|  | Health visitor | 28 | 26.4 | 0.51 | (0.13, 2.01) | 0.86 | (0.16, 4.66) | 1.04 | (0.32, 3.38) |
| **Employer** | |  |  |  |  |  |  |  |  |
|  | University | 29 | 26.6 | *Reference* | |  |  |  |  |
|  | NHS | 71 | 65.1 | 0.88 | (0.25, 3.08) | 0.26 | (0.03, 2.22) | 0.55 | (0.18, 1.67) |
|  | Local Authority | 9 | 8.3 | 1.28 | (0.12, 13.17) | 0.30 | (0.02, 5.29) | 0.45 | (0.08, 2.47) |
| **Length with organisation** | | | |  |  |  |  |  |  |
|  | 0-5 years | 50 | 43.5 | *Reference* | |  |  |  |  |
|  | 6+ years | 65 | 56.5 | 1.39 | (0.52, 3.74) | 0.40 | (0.10, 1.59) | 1.05 | (0.45, 2.45) |
| **Length as qualified clinician** | | | |  |  |  |  |  |  |
|  | 0-5 years | 25 | 22.7 | *Reference* | |  |  |  |  |
|  | 6-10 years | 25 | 22.7 | 2.72 | (0.61, 12.10) | 0.96 | (0.06, 16.24) | 0.88 | (0.27, 2.93) |
|  | More than 10 years | 60 | 54.6 | 2.28 | (0.72, 7.22) | 0.24 | (0.03, 2.03) | 1.65 | (0.57, 4.74) |
| **Qualification** | |  |  |  |  |  |  |  |  |
|  | Degree | 67 | 61.5 | *Reference* | |  |  |  |  |
|  | Diploma | 14 | 12.8 | 1.15 | (0.22, 5.97) | 1.65 | (0.19, 14.65) | 1.05 | (0.29, 3.78) |
|  | Masters/Doctorate | 28 | 25.7 | 0.67 | (0.22, 2.09) | 0.98 | (0.23, 4.11) | 0.94 | (0.35, 2.56) |
| **Training provider focused on promotion and support for breastfeeding** | | | | | | |  |  |  |
|  | NHS employer | 68 | 60.2 | 3.45* | (1.19, 10.07) | 1.49 | (0.40, 5.48) | 1.77 | (0.75, 4.20) |
|  | UNICEF Train the Trainer | 46 | 40.7 | 3.06# | (0.94, 10.03) | 0.28# | (0.07, 1.13) | 1.01 | (0.42, 2.40) |
|  | UNICEF BFI | 68 | 60.2 | 2.50# | (0.89, 6.99) | 0.17# | (0.02, 1.42) | 1.07 | (0.44, 2.58) |
|  | Educational provider | 36 | 31.9 | 1.00 | (0.34, 2.93) | 1.20 | (0.29, 4.96) | 0.74 | (0.30, 1.81) |
| **How long is it since you completed this training?** | | | | | |  |  |  |  |
|  | 0-12 months | 42 | 38.5 | *Reference* | |  |  |  |  |
|  | More than 12 months | 67 | 61.5 | 0.27# | (0.06, 1.29) | 0.35 | (0.07, 1.72) | 1.16 | (0.48, 2.81) |
| **How often do you engage in continuing professional development activity focused on breastfeeding?** | | | | | | | | | |
|  | Annually | 85 | 80.2 | *Reference* | |  |  |  |  |
|  | Bi-annually | 21 | 19.8 | 0.73 | (0.25, 2.12) | 0.35 | (0.09, 1.37) | 1.06 | (0.34, 3.30) |
| **How often does your role involve educating about and or promotion and support of breastfeeding?** | | | | | | | | | |
|  | Rarely/ Weekly | 45 | 39.1 | *Reference* | |  |  |  |  |
|  | Daily | 70 | 60.9 | 1.20 | (0.44, 3.27) | 1.41 | (0.40, 4.96) | 0.96 | (0.40, 2.30) |
| **What drives your work in promoting and supporting breastfeeding** | | | | | | |  |  |  |
|  | Professional duty | 105 | 91.3 | 5.73* | (1.29, 25.45) | 3.37 | (0.59, 19.22) | 2.81 | (0.65, 12.05) |
|  | UNICEF accreditation | 73 | 63.5 | 3.38* | (1.22, 9.37) | 1.11 | (0.30, 4.06) | 0.93 | (0.38, 2.26) |
|  | Employer guidance | 43 | 37.4 | 0.64 | (0.24, 1.74) | 3.16 | (0.65, 15.40) | 1.42 | (0.59, 3.44) |

*Notes.* Logistic Regression significance level: #p≤0.10; *p≤0.05; **p≤0.01; ***p≤0.001
